# Polyphenol Composition and Antioxidant Activity of *Japonica* Rice Cultivars and Intake Status

**DOI:** 10.3390/foods11233788

**Published:** 2022-11-24

**Authors:** Yichao Ma, Shuang Zhang, Liyan Rong, Zhaoxia Wu, Wentao Sun

**Affiliations:** 1 College of Food Science, Shenyang Agricultural University, 120 Dongling Rd., Shenyang 110866, China; 2 Institute of Plant Nutrition and Environment Resources, Liaoning Academy of Agricultural Sciences, Shenyang 110161, China

**Keywords:** *japonica* rice, antioxidant capacity, bound phenolic, canonical correlation analysis, phenolic acid

## Abstract

*Japonica* rice is produced mainly in northeast China, Japan, and the Korean Peninsula. Polyphenols and flavonoids are the main antioxidants in *japonica* rice. This study reported the polyphenol content and antioxidant activity of nine brown and white *japonica* rice cultivars. The total phenolic and flavonoid contents of brown rice were in the ranges of 241.98–296.76 GAE mg/100 g, and 225.30–276.80 RE mg/100 g, respectively. These values were significantly higher than that of white rice by 118.98–206.06% and 135.0–217%, respectively. The bound fraction from phenolics and flavonoids contributed 41.1–63.6% and 62.22–78.19% of the total phenolic and flavonoid content in brown rice, respectively, while these ranges were 55.5–73.5% and 46.07–66.83% in white rice, respectively. p-Hydroxybenzonic acid was the predominant phenolic acid in *japonica* rice. All four antioxidant capacities of brown rice (DPPH, ABTS, OH, FRAP) were higher by up to 1.68–2.85 times than those of white rice. The PZ21 (Yanfeng 47) *japonica* rice variety has outstanding antioxidant capacity based on the weights of each antioxidant index. According to the differences of functional substances among varieties, it can provide guidance for consumers and theoretical basis for the production of healthy food.

## 1. Introduction

Rice is an important staple food that provides daily nutritional and activity energy requirements for humans, particularly in the Asian region [1]. According to the global rice market supply and demand situation analysis (prospect Institute of Industrial Research), global rice production is approximately 509,677 thousand tons, while the rice consumption is 503,908 tons, of which the rice consumption in Asia is approximately one-half of that in the world (2022). Rice contains special bioactive compounds, such as ferulic acid and hydroxycinnamic acids, which have attracted increasing attention from scientists and consumers [2]. These phenolic compounds have antioxidant and anti-inflammatory properties. They are correlated with reduced risks of various chronic diseases, including heart disease, and mitigation of type 2 diabetes symptoms [3]. These highly bioactive compounds are distributed mainly in the bran layer [4]. Therefore, non-uniform distribution of bioactive compounds could influence functional characteristics in rice [5]. However, there is little discussion regarding how differences in the distribution of bioactive compounds may affect the functional characteristics of brown and white rice.

Brown rice contains the bran layer, while stripping the bran layer through a milling process produces white rice [6]. Ferulic acid and p-coumaric acid are the major compounds found in rice bran. Ferulic acid is distributed more evenly throughout the endosperm compared with p-coumaric acid [7]. Ferulic acid has an antioxidant effect and can play an anti-platelet agglutination role, and p-coumaric acid has a hypolipidemic effect in mice [8]. Brown rice, as a whole grain food, is of great interest to consumers and nutritionists. Currently, there are 22 countries with dietary recommendations, but only 8 of them have a recommended intake of whole grains. The recommended daily intake of cereals in the United States is approximately 170 g/d, of which the recommended intake of whole grains is more than 48 g/d. The recommended intake in China is 50–150 g/d, while it is 90 g/d for men and 70 g/d for women in Sweden. Australia and Turkey have higher recommended intakes of 240 g/d and 300 g/d, respectively. However, although the current dietary structure of residents consists mainly of cereal, refined rice serves mostly as the staple food, and less than 20% of adults can meet the whole grain intake of 50 g/d. However, the dietary levels of phenolic acids induced by the consumption of three ounces of brown and white rice are unknown.

Phenolic acids are a class of organic acids containing phenolic rings. These include gallic acid, p-hydroxybenzoic acid, ferulic acid, and other compounds containing phenolic rings. Phenolic acids in rice are present in free and bound forms. Free phenolic compounds, such as cinnamic acid and gallic acid, can play a positive role against the development of colon cancer and some chronic diseases in the digestive tract. Bound phenolic compounds, such as ferulic, coumaric, and caffeic acid, may be hydrolyzed in the large intestine by intestinal enzymes, releasing them from bound macromolecules [9,10,11]. However, the previous literature studying changes in the free and bound forms of phytochemicals in rice did not consider the impact of rice varieties.

The antioxidant activity of grain is promoted by different phytochemical substances in vitro [12]. Polyphenols are the main antioxidants in rice, while other bioactive compounds, such as phytosterols, also have antioxidant properties [13]. These bioactive antioxidant substances act as a preventive and protective mechanism against chronic diseases caused by oxidative damage due to excessive free radical production in living organisms [14]. There are many methods to measure antioxidant activity of these substances in vitro, such as DPPH, ABTS, PSC, and ORAC, which are based on different antioxidant mechanisms [15]. However, these methods evaluate only the antioxidant activity of food from a single oxidation mechanism and do not comprehensively consider the overall antioxidant activity. Multivariate statistical analysis makes it possible to analyze the relationship between phenolic compounds and antioxidant activity in foods. For example, canonical correlation analysis calculates the correlation of aggregate variable pairs to reflect the overall correlation of two groups of indicators, while principal component analysis mainly carries out dimension reduction analysis for various indicators, as well as cluster analysis and the entropy weight method [7]. Canonical correlation analysis has the advantage of integrating the relationship analysis between two groups of data, while other analyses show only the relationships among multiple indicators, rather than the analysis among groups. Previous studies very rarely analyzed the bioactivity and antioxidant capacity of cereals by combining multiple multivariate statistical analyses.

With rapid nutrition transition, dietary intake and nutritional status has gained increasingly more attention in the past decades. Therefore, it is necessary to thoroughly investigate the bioactive substances and antioxidants of brown and white rice from different *japonica* varieties to gain a deeper understanding of their effects on human health.

This study aimed to determine the polyphenol and flavonoid content in free and bound parts of brown and white rice and the antioxidant activities in nine *japonica* rice varieties. The correlation between active substances and antioxidant activity was analyzed. Canonical correlation analysis and a network correlation graph were used to analyze the indexes greatly contributing to the antioxidant activity of different rice varieties, and the independent weight was used to comprehensively evaluate antioxidant capacity. This offers a theoretical basis for the development of functional rice. At the same time, the total phenolic acid intake of different varieties of brown rice in different age groups was evaluated. The study also predicted dietary phenolic acid intake associated with 3 ounces of brown rice as a white rice substitute. It will be helpful to further study the biological activity and health mechanism of functional substances in brown rice, and it is of great significance to study the complex relationship between diet and health.

## 2. Materials and Methods

### 2.1. Raw Material

The test area of the present study was conducted at Liaohe Delta, Liaoning Province, China (122°14′17″ N, 41°9′31″ E). The seedlings were transplanted on May 25 and were harvested on October 8 in 2020.This experiment involved nine *japonica* rice varieties. The mature period of *japonica* rice was medium and newest maturing. The physical indexes of nine *japonica* rice varieties planted in this area are shown in Table 1.

### 2.2. Color Determination

A Minolta spectrophotometer CM-3500d colorimeter (Minolta Co., Ltd., Osaka, Japan) with Spectra Magic version 3.6 software was used to measure the color of rice samples. The color was expressed using the *L*, a*, and b* color space coordinates, where *L* represents lightness, a* represents redness to greenness, and b* represents yellowness to blueness. Yellowness (YI) and whiteness (YI) were used to describe the color difference of rice.

(1) $$\mathrm{YI}=142.86\times \frac{b}{L}$$

(2) $$\mathrm{WI}=100-\sqrt{{\left(100-L\right)}^{2}+K_{1}\;\left(a^{2}+b^{2}\right)}$$

### 2.3. Determination of Total Phenolic

Total phenolic content (TPC) was determined by Folin-Ciocalteu colorimetric method performed previously [16]. Briefly,100 μL of sample extractor was treated with 100 μL of Folin-Ciocalteu reagent for 6 min. Afterward, the mixture was alkalinized with 1 mL of 7% Na_2_CO_3_. After being kept in the dark for 90 min, a microplate photometer was used to measure the absorbance of each sample at 760 nm, which was repeated three times for each extract. Standard gallic acid (GA) curve was used to calculate content. TPC of each sample was presented as mg GAE per 100 g dry weight.

### 2.4. Determination of Flavonoid Content

Currently, the determination of TFC was depended on the aluminium chloride colorimetric method described by [17]. Briefly, a 50 μL supernatant was mixed with 100 μL distilled water. Then, 5% NaNO_2_ was added into the mixture and incubated for 5 min. Subsequently, 10% AlCl_3_ 6H_2_O solution was drawn and added to the mixture for incubation for 3 min. Finally, 60 μL 4%NaOH was added to the termination reaction. The samples were read at 510 nm. Absolute methanol was used as the control, while a standard rutin curve was used to calculate the content of TFC. Results were recorded as mg of RE/100 g DW.

### 2.5. Determination of Phenolic Acid Content

The determination of phenolic acid content was performed on HPLC as described previously [12]. Quantitative analysis of phenolic acid compounds was performed by HPLC using an Agilent 1200 HPLC system (Waldbronn, Germany) and a Zorbox SB-C18 column (250 × 4.6 mm, Agilent Technologies, Palo Alto, CA, USA). The standards (syringic acid, p-coumaric acid, caffeic acid, sinapic acid, ferulic acid, and p-hydroxybenzoic acid) of purity were greater than 97%, purchased from Sigma-Aldrich Corporation. To configure the phenolic acid standard solution (1 mg/ mL), the standard working liquid of phenolic acid mixture was prepared by gradient dilution method. The mobile phase was made up with 0.05% trifluoroacetic acid in water (A) and methanol (B). The gradient elution program was set as follows: 0–10 min, 10% B; 10–20 min, 10–25% B; 20–30 min, 25–35% B; 30–40 min, 35–45% B; 40–50 min, 45–55% B; 50–60 min, 55–100% B; and 60–65 min, 100–10% B. Ten microliters of sample extract was injected and flowed at a speed of 0.8 mL/min. The content of 6 phenolic acids was calculated by the peak area ratio method. The phenolics contents were represented as mg/100 g DW.

### 2.6. Determination of Antioxidant Activity

#### 2.6.1. Determination of DPPH Radical Scavenging Activity

The method by [18], was used with slight modifications to assess DPPH. The mixtures were shaken vigorously, and the sample was taken then incubated for 30 min in the dark. Mixture was measured at 517 nm.

(3) $$\mathrm{DPPH}\;\mathrm{radical}\;\mathrm{scavenging}\;\mathrm{effect}\;\left(\%\right)=1-\frac{A_{\mathrm{sample}}-A_{\mathrm{background}}}{A_{\mathrm{control}}}\times 100\%$$

where A_sample_, A_control_, and A_background_ refer to sample (sample and DPPH), control (without sample), and background (without sample), respectively.

#### 2.6.2. Determination of Hydroxyl Radical-Scavenging Activity

The method by [18], was used with slight modifications to assess activity of each extract. One millilitre of anhydrous ethanol solution (with 1.865 mmol/L phenanthroline monohydrate) and 1 mL phosphate buffer (pH 7) were added to 2 mL of the samples. The mixtures were then vigorously shaken, and 1.0 mL of 1.865 mmol/L FeSO_4_ solution was added. The absorbance was measured at 536 nm.

(4) $$\mathrm{Hydroxyl}\;\mathrm{radical}-\mathrm{scavenging}\;\mathrm{effect}\;\left(\%\right)=\frac{A_{S}-A_{n}}{A_{b}-A_{n}}\times 100\%$$

where A_n_, A_s_, and A_b_ refer to the absorbance of the control (water replaced H_2_O_2_), sample, and background solution (without sample), respectively.

#### 2.6.3. ABTS Free-Radical-Scavenging Activity

ABTS method was performed according to [19] with slight modifications. The working solution (ABTS solution and potassium persulfate solution) was required to reach 0.7 ± 0.02 at 734 nm. Then, 0.1 mL sample solution was mixed with 1.4 mL ABTS solution, which required 1 h at 25 °C to incubate. The absorbance was read at 734 nm.

(5) $$\mathrm{ABTS}\;\mathrm{radical}-\mathrm{scavenging}\;\mathrm{activity}\;\left(\%\right)=1-\frac{A_{\mathrm{sample}}}{A_{\mathrm{control}}}\times 100\%$$

A_sample_ and A_control_ are the absorbance of the extract and control, respectively.

#### 2.6.4. Ferric-Reducing Antioxidant Power Assay (FRAP)

FRAP was used by the method of [19], with slight modifications. The FRAP working solution (2 mL) was added to 50 μL extraction solution(or control). Absorbance was read at 593 nm. A standard Vc curve was used to calculate content. The FRAP of the sample was expressed as VCEAD.

### 2.7. Estimation of Phenolic Acids Dietary Intake

PDI is phenolic acid daily intake, PCF is phenolic acid concentration in samples, and FDI is food daily intake. The data came from “Survey on the Status of Nutrition and Health of Chinese People” [20]. The PDI was calculated as follows:

(6) $$\mathrm{PDI}=\overset{n}{\underset{K=0}{\sum }}\mathrm{PCF}\times \mathrm{FDI}$$

### 2.8. Statistical Analyses

All measurements in this study were presented as means ± standard deviations. Each antioxidant activity assay was carried out three times from the same extracts in order to determine their reproducibility. Statistical differences and principal component analysis were analyzed with SPSS 25 (SPSS Inc., Chicago, IL, USA) [21]. Canonical correspondence analysis and networks were conducted with Origin software.

## 3. Results

### 3.1. Color Parameter Analysis

Color characteristics are often a very useful way to identify different rice varieties [3]. The color parameters, yellowness values (YI) and whiteness values (WI), of nine *japonica* rice varieties are shown in Table 2. YI, WI, and the a* value of nine brown rice varieties were significantly different (*p* < 0.05), while the *L* and b* values of brown rice varieties did not significantly vary (*p* > 0.05). The yellowness value of PZ13 was higher than that of PZ6, PZ8, PZ15, and PZ19, while the whiteness values of PZ8 (59.45) and PZ15 (59.19) in brown rice were slightly higher than those of the other varieties. The differences in color among nine *japonica rice* varieties grown in the same area could be due to varietal genotypes, where there are differences in regulatory genes responsible for seed coloration. There are 26 genes associated with rice color in plant growth, 15 of which are involved in the coloration of other active compounds, including anthocyanins and flavonoids. There were no significant differences in the b* value and yellowness value of the different white rice varieties (*p* > 0.05). This may be due to the absence of the outer bran layer from white rice, as the bran color affects the yellowness value. Meanwhile, the *L* value, a* value, and whiteness value of different white rice varieties showed significant differences (*p* < 0.05). The *L* value of PZ21 was 70.92, which was much higher than that the other varieties and had a brighter color. The a* values of PZ6, PZ10, and PZ21 were negative, indicating a light green color, which was significantly different from the a* values of PZ9, PZ13, and PZ15. PZ21 had the highest whiteness value (68.11), and the whiteness values of different white rice varieties were significantly higher than those of brown rice, while the brown rice yellowness value was much higher than that of white rice, likely due to the fact that brown rice retains the bran layer.

### 3.2. The Phenolic Content of Japonica Rice Varieties

**Figure 1:**
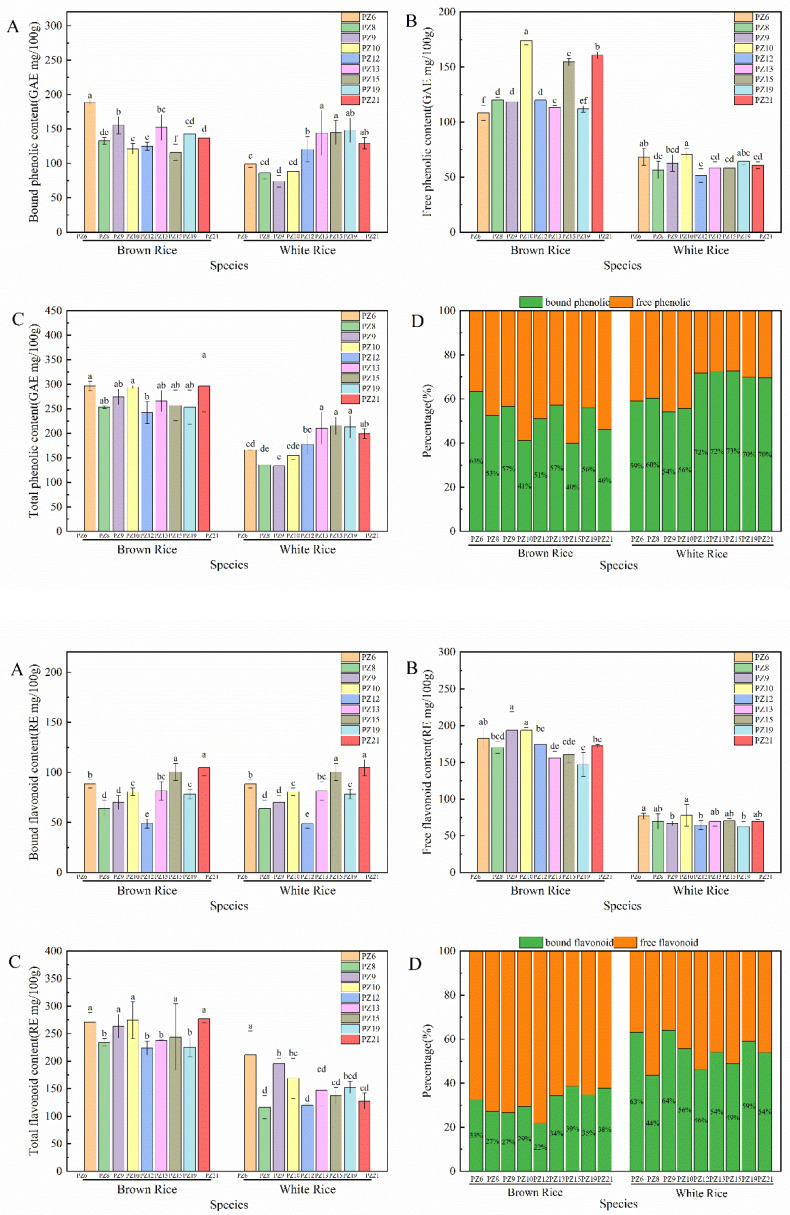
Bound, free, and total phenolic and flavonoid content of *japonica* rice varieties ((**A**): Bound phenolic and flavonoid; (**B**): Free phenolic and flavonoid; (**C**): Total phenolic and flavonoid). Percentage bound phenolic and flavonoid content out of the total content (**D**). Values of each bar with lowercase letters in common are significantly different (*p* < 0.05).

The TPC of brown rice and white rice of different *japonica* rice varieties were significantly different (*p* < 0.05) (Figure 1). The TPC measured in brown rice was significantly higher (118.98–206.06%) than that of white rice. Brown rice GAE levels were the highest in PZ21 (296.76 mg/100 g), while the lowest content was found in PZ12 (241.98 GAE mg/100 g). The highest and lowest contents of GAE were found in PZ15 (215.06 mg/100 g) and PZ9 (133.08 mg/100 g). The differences in total phenolic content among varieties may be due to genotypic differences. It is noteworthy that the TPC of PZ15 was the highest among the white rice, but it was only 119.47% higher than the lowest white rice varieties. This may indicate that milling during rice processing has different effects on the active compounds of different varieties. Meanwhile, the difference between the brown and white rice rate in different varieties may be due to the inconsistent trend of total phenolic content in different *japonica* varieties.

The free polyphenol contents from distinct *japonica* rice varieties were significantly different (*p* < 0.05). The range of free polyphenol content in brown rice was 108.36–173.99 GAE mg/100 g, and the range of free polyphenol content in white rice was 51.36–70.75 GAE mg/100 g. The free polyphenol content in all brown rice was 1.5 times higher than that in white rice, of which the content in brown rice PZ21 was 264.84% higher than that in white rice. The free polyphenol content of brown and white rice of PZ10 was the highest, while the lowest content was found in brown rice from PZ6 and white rice from PZ12.

The bound polyphenol contents from different *japonica* varieties were significantly different (*p* < 0.05). The bound polyphenol content in brown rice was in the range of 112.83–188.48 GAE mg/100 g. White rice had 0.48–1.28 times higher bound polyphenol content than brown rice. Notably, brown rice of PZ15 and PZ19 contained below 21.14% and 0.04% of the bound phenol content compared with white rice, respectively. However, brown rice of PZ9 contained 2.1 times more bound phenols than white rice. Milling brown rice into white rice had a greater effect on the bound phenols, and different varieties were affected by the degree of milling. Phenolic compounds are located mostly in the external rice grain layer. The total phenolic content was highest in the bran fraction in distinct rice genotypes.

The bound phenols contributed 41.1–63.6% of TPC in brown rice and 55.5–73.5% in white rice (Figure 1D). White rice had a much higher percentage of bound phenols out of the total phenol content compared with that in brown.

### 3.3. Flavonoid Content of Japonica Rice Varieties

The TFC of brown and white rice of different *japonica* rice varieties were significantly different (Figure 1; *p* < 0.05). The TFC of brown rice was in the range of 225.30–276.80 RE mg/100 g, while that of white rice was in the range of 119.81–211.03 RE mg/100 g: brown rice had 135.0%-217% higher total flavonoid content than that of white rice. Among them, the difference between brown and white rice from PZ9 was smaller. The different trends in TFC and TPC were variant in the nine varieties, likely because the distribution sites of total phenols and flavonoids in rice were influenced by the genotype.

In the nine *japonica* varieties, the free flavonoid contents of brown and white rice were significantly different (*p* < 0.05). In brown rice, it was in the range of 146.98–193.65 RE mg/100 g, with PZ9 and PZ10 having higher contents, and the free flavonoid content in white rice was in the range of 62.49–77.92 RE mg/100 g. The highest increase in the free content of brown rice compared with white rice was observed in PZ9 (289.60%). The free flavonoid content and phenol content of brown and white rice of PZ10 showed similar trends, both of which were the highest among all varieties.

There were significant differences in the bound flavonoid content in brown and white rice of the nine *japonica* varieties (*p* < 0.05). The bound flavonoid contents in brown and white rice were in the range of 48.74–104.37 RE mg/100 g and 53.51–141.04 RE mg/100 g, respectively. The bound flavonoid content in brown rice varied by 0.59–1.50 times compared with that in white rice for the same varieties, while PZ6, PZ9, PZ10, PZ12, and PZ19 all had lower bound flavonoid content in brown rice compared with that of white rice. The bound content in brown rice was 0.48–1.28 times higher than white rice. Notably, the bound flavonoid content in brown rice of PZ15 and PZ19 was lower than the content in white rice by 21.14% and 0.04%, respectively. However, brown rice of PZ9 contained 2.1 times more bound phenol than that of white rice.

**Figure 2:**
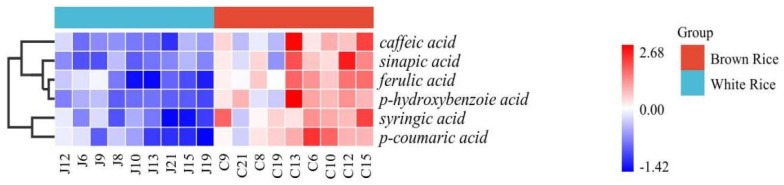

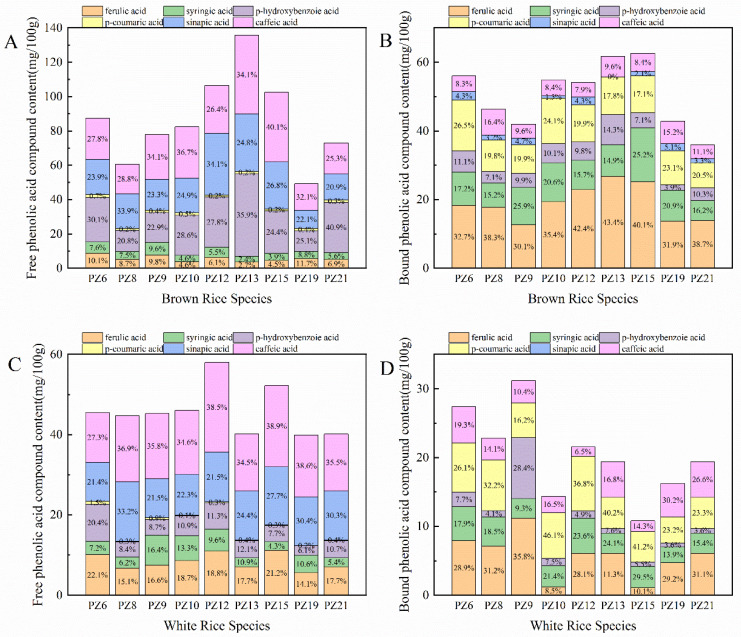
Heat map showing the content of six phenolic acids in brown and white rice and the percentage contribution to the total content of each phenolic compound. Free (**A**,**C**) and bound (**B**,**D**) phenolic compound content of brown and white rice, respectively. Values of each bar with lowercase letters in common are significantly different (*p* < 0.05).

The percentages of bound flavonoid content to total flavonoid content in different varieties are shown in Figure 2D. The bound flavonoid content in brown rice was lower than that of free flavonoid content, with the highest percentage of bound form to total flavonoids observed in brown rice from PZ15 (39%). However, the bound flavonoid content in white rice was higher than 44% of the total flavonoid content in all cases, while the contribution of bound flavonoids to total flavonoids in white rice of PZ9 reached 64%. There were large differences in free and bound flavonoid contents between brown and white rice of the same variety. The trend of flavonoid content of different varieties was similar to that of total phenolic content.

### 3.4. Phenolic Acid Content of Japonica Rice Varieties

The phenolic acid composition and content significantly varied among varieties, while the phenolic acid content in each brown rice was significantly higher than that in white rice (*p* < 0.05) (Figure 2). Liao Xing 1 brown rice (C13) contained relatively high contents of caffeic acid, sinapic acid (a hydroxycinnamic acid), ferulic acid, and p-hydroxybenzoic acid, while the overall contents of six phenolic acids of Yanfeng 47 (C21), Liao *Japonica* 1305 (C8), and Shen Nong 9816 (C19) were lower than those of other brown rice varieties. Meanwhile, Liao *Japonica* 419 white rice (J12) had relatively higher overall content of each phenolic acid. p-Hydroxybenzoic acid content was 13.71–57.07 mg/kg, and this was the main phenolic acid component, while p-hydroxybenzoic acid in white rice was 2.99–12.78 mg/kg. The brown rice p-hydroxybenzoic acid content in PZ13 was more than eight times that of white rice. Ferulic acid is an important phenolic acid component in rice; its content was 18.85–29.71 mg/kg in brown rice and 9.54–18.66 mg/kg in white rice. The white rice contents of these same two acids were 5.0–67.4% and 7.4–65.2% lower than those in brown rice, respectively. Both syringic acid and p-coumaric acid showed similar trends in different varieties, with less variation in the content of p-coumaric acid in different varieties. Eugenic acid content in brown rice was in the range of 10.02–19.79 mg/kg, which was 1.24–2.61 times higher than the content of white rice. p-coumaric acid was lowest in brown rice with 7.60 mg/kg in C21 and the lowest in semolina with 3.86 mg/kg in J19. The content of ferulic acid and sinapic acid from the nine varieties was significant (*p* < 0.05). The trend of ferulic acid content in the nine varieties was similar to that of sinapic acid. The changes in the content of p-hydroxybenzoic acid and caffeic acid of the nine varieties were similar. Moreover, PZ19 had the lowest content of syringic acid, p-hydroxybenzoic acid, p-coumaric acid, and caffeic acid.

p-Hydroxybenzoic acid, sinapic acid, and caffeic acid were found mainly in the free fraction of the nine *japonica* rice varieties, while ferulic acid, syringic acid, and p-coumaric acid existed mainly in the bound fraction. p-Hydroxybenzoic acid is the main phenolic acid in brown rice, followed by ferulic acid. The total content of p-Hydroxybenzoic and ferulic acids accounted for approximately 50% of the total content of six phenolic acids in different varieties. The proportion of six phenolic acids in PZ6, PZ8, and PZ9 were similar, and each phenolic acid accounted for 8–22% of the total phenolic acid content. PZ10 and PZ12 had similar proportions of six phenolic acids. However, the content of p-hydroxybenzoic acid in varieties PZ13 and PZ21 was much higher than that of other varieties, accounting for 38% and 29%, respectively.

### 3.5. Differences in Antioxidant Activity of Nine Japonica Rice Varieties

**Figure 3:**
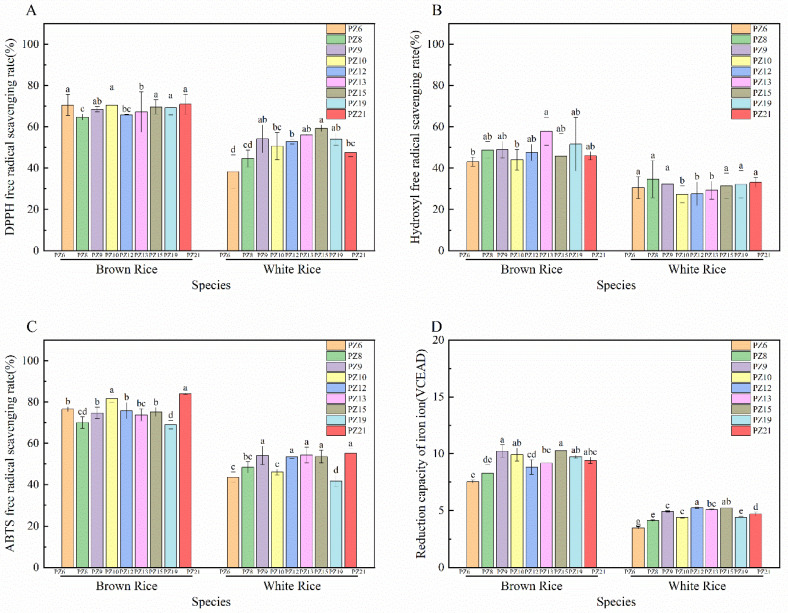

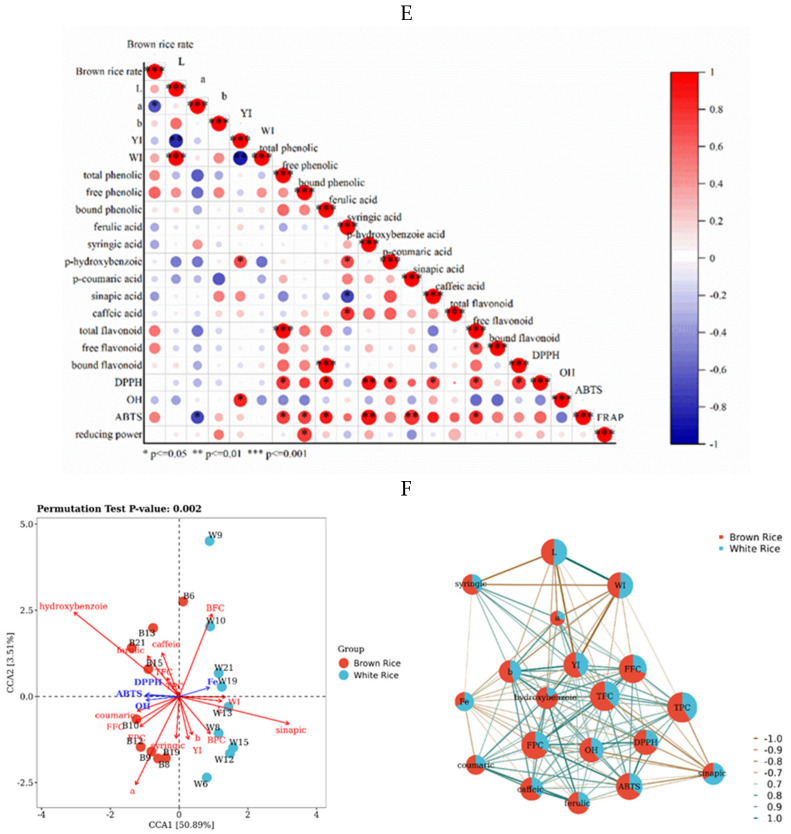
Antioxidant activity of nine *japonica* rice varieties ((**A**): DPPH, (**B**): OH, (**C**): ABTS, (**D**): FRAP). Correlation between polyphenol composition and antioxidant activity (**E**). Canonical corresponence analysis and networks (**F**). Values of each bar with no letters in common are significantly different (*p* < 0.05).

The mechanisms of action of antioxidant capacity are different. Therefore, DPPH, ABTS, hydroxyl radical-scavenging capacity, and FRAP were selected to evaluate the antioxidant activity of nine *japonica* rice varieties (Figure 3).

The DPPH radical-scavenging rate significantly varied in brown rice (64.72–70.92%, *p* < 0.05). Brown rice of PZ8 had the lowest antioxidant ability to scavenge DPPH radicals and was significantly different from other varieties (*p* < 0.05). The coefficient of variation among different varieties was 3.22%. Meanwhile, the scavenging rate of DPPH free radicals in white rice was 17.74% lower than that in brown rice, on average. PZ13 had the highest scavenging rate (68.28%), while PZ6 had the lowest scavenging rate (31.22%). The DPPH antioxidant activities of different white rice varieties were significantly different (*p* < 0.05).

The hydroxyl radical-scavenging rate was lower than 60% in all nine brown rice varieties, with a range variation of 43.06–57.87% and a coefficient of variation of 7.87% among brown rice varieties. The scavenging rates of PZ6 and PZ10 were lower, 43.06% and 44.02%, respectively, while the hydroxyl radical-scavenging ability of the other brown rice varieties did not significantly vary (*p* > 0.05). The hydroxyl radical-scavenging rate of brown rice was approximately 12.49–28.56% higher than that of white rice. Among them, white rice (PZ8) had the highest content of 34.62%. The hydroxyl radical antioxidant capacities of all white rice was below 35%, and the overall scavenging rate was lower than that of DPPH radicals. The coefficient of variation of hydroxyl radical-scavenging ability among the nine white rice varieties was 5.93%.

The ABTS radical-scavenging rate in brown rice significantly varied from the nine *japonica* varieties (69.02–84.08%, *p* < 0.05), and the coefficient of variation was 6.41%. Among them, PZ21 had the highest antioxidant activity. The scavenging rate of ABTS radicals in brown rice was 1.36–1.77 times higher than that in white rice (25.61% higher, on average), and the highest scavenging ability of 55.09% was found in PZ21. The coefficient of variation among the nine white rice samples was 10.37%. The difference in scavenging rates of different white rice varieties was greater than that of brown rice.

The range of variation of FRAP was significantly different in brown rice among the nine *japonica* varieties (7.54–10.25 VCEAD) (*p* < 0.05), and the coefficient of variation was 2.62%. Among them, PZ9 and PZ15 had higher reducing ability. The reducing ability of brown rice was 1.68–2.26 times higher than that of white rice, with the highest reducing ability of 5.23 VCEAD in white rice of PZ12. There was a significant difference in reducing ability among white rice from different rice varieties (*p* < 0.05).

All four antioxidant capacities in brown rice were higher than those of white rice. The hydroxyl radical and FRAP differed more between brown and white rice. The greater variation of different antioxidant capacities among the same varieties may be due to the different mechanisms of antioxidant action. The independent weighting method was used to calculate the weights of the four antioxidant methods to better understand the whole antioxidant capacity of brown and white rice from different *japonica* varieties (Table 3). The antioxidant levels of the nine varieties were comprehensively calculated, and ranking was performed on the basis of the weights of each antioxidant index. Brown rice has a higher FRAP ability, while white rice had stronger hydroxyl radical-scavenging ability. The general antioxidant capacities of PZ21 (Yanfeng 47) and PZ13 were the highest among brown rice and white rice, respectively.

### 3.6. Correlation between Polyphenol Composition and Antioxidant Activity

Correlation analysis was used to assess the relationship between color characteristics, polyphenol composition, and antioxidant activity of different *japonica* rice species (Figure 3). The correlation between FRAP and most of the polyphenols’ content was greater than 0.5 in all brown and white rice varieties, but there was no significant difference (*p* > 0.05). Antioxidant activity (DPPH) positively correlated with free phenols (0.7717), free flavonoids (0.7411), ferulic acid (0.8852), p-hydroxybenzoic acid (0.9228), p-coumaric acid (0.8222), and caffeic acid (0.7376). This suggests that these compositions are the main contributors to scavenge DPPH radical. There was a high positive correlation between the contents of bound phenols and bound flavonoids (*p* < 0.01). A significant negative correlation between the a* value and ABTS indicated that the color of brown rice may affect ABTS radical antioxidant activity. ABTS was positively correlated with free phenols (0.7405), bound phenols (0.7401), free flavonoids (0.7838), ferulic acid (0.8619), p-hydroxybenzoic acid (0.9066), and sinapic acid (0.7815). However, there was a negative correlation between hydroxyl radical scavenging ability and the following: whiteness, total phenols, free phenols, total flavonoids, and free flavonoid contents.

### 3.7. Canonical Correspondence Analysis and Networks

Multivariate statistical analysis was performed through canonical correlation analysis to investigate the relationships among bioactive components (polyphenols, flavonoids, and phenolic acids) and antioxidant activities (DPPH, OH, ABTS, FRAP) (Figure 3). p-Hydroxybenzoic acid had the most significant effect on brown rice from different rice varieties. Bound phenolics and flavonoids had a greater impact on white rice. White rice was affected mainly by bound phenolics and flavonoids. The nine different *japonica* rice varieties of brown rice can be divided into two categories: PZ8, PZ9, PZ10, PZ12, and PZ19, which are affected mainly by syringic acid and coumaric acid; and PZ13, PZ15, and PZ21, which are affected mainly by caffeic acid and ferulic acid. PZ21 and PZ13 of brown rice have higher antioxidant activities, while PZ19 and PZ21 of white rice have higher antioxidant activities, according to the distance between samples and antioxidant index factors. In conclusion, PZ21 (Yanfeng 47) has outstanding antioxidant capacity.

### 3.8. Intake from Brown and White Rice of Phenolic Acids

The daily intakes of phenolic, flavonoids, and phenolic acids from white and brown rice are shown in Table 4. If only white rice were consumed, the most abundant content by standard person per day was flavonoid, which occupied 31.8–48.2%. If three ounces of white rice was replaced with brown rice, the diversities of phenolic, flavonoids, and phenolic acids all increased. For phenolic acids, the intake would increase by approximately 1.08–2.34 times. For phenolic and flavonoid, the highest increases were 1.43 times and 1.51 times, respectively. It is worth noting that the daily recommended intake of white rice is two ounces higher than that of brown rice, and the total active substance content of brown rice is higher than that of white rice. The contents of phenols, flavonoids, and phenolic acids that can be provided to people in daily life are different among different varieties. Therefore, different groups of people can choose different varieties of brown rice to meet their daily dietary intake.

## 4. Discussion

### 4.1. Differences in Free and Bound Form Components in Different Japonica Rice Varieties

Phenolic compounds occur generally as free and bound forms [22]. Bound phenolics contribute approximately 20% of the total phenolic content that exists in fruits and vegetables, whereas the proportion is often as high as 60–80% in whole grains [23]. In this study, bound phenolics comprised more than 40% and 56% of the total phenolics in brown and white rice, respectively, from the nine *japonica* varieties. Meanwhile, bound flavonoids comprised at least 61% and 44% of total flavonoid phenolic content in brown and white rice, respectively.

Bound phenols contribute 60% of the total phenolic content in whole grain rice [24], with free phenolics contributing 38% of the total amount [25]. The results are consistent with the trend of this experiment: phenols and flavones in *japonica* rice were distributed mainly in bound form. However, [8] showed that the bound form provides 23.5–29.7% phenols and 31.7–47.5% flavonoids of the total content in white rice. The difference in bioactive content between this study and previous studies may be related to the different varieties analyzed. These bound forms may exert a healthy local benefit by releasing phenolics through microflora [26].

Bound phenolics are potent antioxidants and contain prebiotic activity in bran [10]. Brown rice may display a better health benefit considering the difference in total phenols and flavonoids between brown and white rice. However, total polyphenol content in white rice is still worthy of attention because it is derived from 75% of brown rice’s dry weight, and overall, white rice contributes to good health. Therefore, bound phenolics are a non-negligible health benefits in *japonica* rice. PZ6 and PZ21 had a higher proportion of bound form among the nine *japonica* rice varieties, and they could be considered for the development of functional food.

### 4.2. Antioxidant Activities of Different Japonica Rice Varieties

This study determined the variation trend of bioactive components of different varieties of brown and white rice. This is expected to improve the nutritional value and antioxidant activity of *japonica* rice. There was a linear association between the level of phenolic compounds and the antioxidant capacity of brown and white rice. Bound phenolic compounds were the main antioxidant components in *japonica* rice. Phenolic components have antioxidant activity and possible health benefits. Phenolic acids are the most common phytochemicals with antioxidant activity in cereal crops, and the phenolic acid composition in plants is divided into two main groups: hydroxycinnamic acid and hydroxybenzoic acid [27]. Pinent [28] reported that the interactions among phenolic acids can affect antioxidant capacity.

The different antioxidant activities of the nine rice varieties in this study may be related to the phenolic acid structure. p-Hydroxybenzoic acid contributed most of the antioxidant capacity of *japonica* rice. Ferulic, caffeic, and p-coumaric acid were highly positively correlated and were highly correlated with DPPH (0.8852, 0.8229, and 0.7376, respectively) and ABTS (0.8618, 0.7815, and 0.8734, respectively). This may be due to the structural similarity of the three phenolic acids, which contain a hydroxyl group on the benzene ring. They may be hydrogen-donating antioxidants against nitrogen radicals [29]. This result was consistent with previous work by [30], showing a high correlation between phenolic acid content and DPPH antioxidant activity (0.781). Syringic acid and p-hydroxybenzoic acid are hydroxybenzoic acids because of their carboxyl group and stable structure; therefore, they have a positive effect on antioxidant capacity of *japonica* rice. However, syringic acid and sinapic acid both have two methoxy groups, which has little effect on *japonica* rice varieties. In addition, sinapic acid showed a negative correlation with syringic acid (−0.7037). Sinapic acid and syringic acid had opposite antioxidant activities in *japonica* rice. The different antioxidant capacities between brown and white rice may be caused by the different proportions and structure of phenolic acids among different varieties.

The antioxidant activity of rice is related to the content of phenols, flavonoids, and phenolic acids, and is also affected by the appearance and color characteristics of rice. Antioxidant ability is positively correlated with free phenolic content in different pigmented rice genotypes [31]. Rice with a blacker bran color exhibits higher variable extractable phenolic content. Meanwhile, there is a frequent correlation between antiradical efficiency and phenolic content. There was a significant negative correlation between a* and ABTS (−0.7117) and a positive correlation between YI and OH (0.8504).

Growing conditions and climates can also impact phenolic compositions and antioxidant activity. Thus, nine *japonica* rice varieties planted in the same area and period were selected in this experiment.

## 5. Conclusions

Brown rice contains a large number of phenolic compounds and exhibited high antioxidant activity. Bound fractions contributed to the majority of antioxidant activity in brown and white rice. There was a linear association between the level of phenolic compounds and the antioxidant capacity of brown and white rice. Antioxidant activity is related to phenols, flavonoids, and phenolic acids and is affected by its appearance and color characteristics. Non-colored rice may also reflect the difference in antioxidant activity of different varieties according to the YI color value. Independent weight analysis showed that the comprehensive antioxidant capacity of Yanfeng 47 (PZ21) was better than that of the other varieties, which could be used as a reference for subsequent agricultural extension studies. The differences in antioxidant activity among rice varieties were significantly influenced by genotypes.

## Figures and Tables

**Table 1:** Nine *japonica* rice varieties.

Sample	Name	Brown Rice Rate (%)	White Rice Rate (%)	Whole White Rice Rate (%)	Growth Period (Days)
PZ6	Salt *japonica* 218	81.7	72.9	69.1	161
PZ8	Liaoning *japonica* 1305	83.6	75.9	74.8	158
PZ9	ShenNong 265	82.4	75.1	63.3	155
PZ10	Salt *japonica* 456	83.8	74.5	70.6	163
PZ12	Liaoning *japonica* 419	81.5	71.9	66.8	159
PZ13	Liao star 1	82.1	74.3	65.6	157
PZ15	1804	81.8	73.6	70.1	154
PZ19	ShenNong 9816	80.9	72.1	69.4	157
PZ21	Yanfeng 47	83.4	75.1	73.6	143

**Table 2:** Color analysis of different brown rice and white rice *japonica* variety.

	Variety	*L*	a*	b*	YI	WI
Brownrice	PZ6	62.71 ± 2.06 ^a^	2.92 ± 0.48 ^ab^	21.89 ± 0.55 ^a^	49.80 ± 1.19 ^a^	56.66 ± 1.55 ^a^
	PZ8	66.39 ± 2.26 ^a^	3.04 ± 0.52 ^ab^	22.49 ± 1.46 ^a^	48.32 ± 3.24 ^a^	59.45 ± 1.86 ^b^
	PZ9	62.90 ± 0.57 ^a^	3.48 ± 0.18 ^b^	22.43 ± 0.51 ^a^	50.89 ± 0.84 ^ab^	56.50 ± 0.31 ^a^
	PZ10	63.75 ± 2.33 ^a^	2.11 ± 0.88 ^a^	22.38 ± 0.65 ^a^	50.09 ± 2.03 ^ab^	57.35 ± 1.95 ^a^
	PZ12	64.09 ± 2.20 ^a^	3.20 ± 0.29 ^ab^	22.84 ± 1.15 ^a^	50.85 ± 0.87 ^ab^	57.32 ± 1.24 ^a^
	PZ13	61.92 ± 3.67 ^a^	2.47 ± 1.11 ^ab^	22.46 ± 0.32 ^a^	51.76 ± 3.68 ^b^	55.72 ± 3.21 ^a^
	PZ15	66.34 ± 1.52 ^a^	3.27 ± 0.52 ^ab^	22.84 ± 0.49 ^a^	49.12 ± 2.11 ^a^	59.19 ± 1.54 ^b^
	PZ19	63.69 ± 3.27 ^a^	3.58 ± 0.31 ^b^	22.28 ± 1.93 ^a^	49.91 ± 3.76 ^a^	57.25 ± 2.43 ^a^
	PZ21	65.16 ± 2.11 ^a^	2.40 ± 0.61 ^ab^	22.93 ± 0.52 ^a^	50.22 ± 2.58 ^ab^	58.22 ± 1.96 ^ab^
		*L*	a*	b*	YI	WI
Whiterice	PZ6	66.99 ± 2.37 ^ab^	−0.15 ± 0.50 ^a^	14.53 ± 1.85 ^a^	30.95 ± 2.23 ^a^	63.93 ± 2.31 ^b^
	PZ8	68.91 ± 1.03 ^ab^	0.48 ± 0.11 ^b^	15.36 ± 1.00 ^a^	31.79 ± 1.71 ^a^	65.32 ± 0.63 ^bc^
	PZ9	66.55 ± 2.23 ^a^	1.37 ± 0.28 ^c^	17.51 ± 0.28 ^a^	37.55 ± 1.84 ^a^	62.21 ± 2.11 ^ab^
	PZ10	67.53 ± 2.91 ^ab^	−0.41 ± 0.47 ^a^	15.25 ± 0.89 ^a^	32.23 ± 2.94 ^a^	64.12 ± 2.86 ^b^
	PZ12	65.90 ± 1.52 ^a^	1.06 ± 0.33 ^bc^	18.28 ± 0.44 ^a^	39.57 ± 1.57 ^a^	61.30 ± 1.45 ^a^
	PZ13	66.55 ± 0.73 ^a^	1.46 ± 0.12 ^c^	18.51 ± 1.10 ^a^	39.68 ± 2.12 ^a^	61.75 ± 0.50 ^ab^
	PZ15	68.63 ± 1.45 ^ab^	1.33 ± 0.52 ^c^	17.46 ± 1.98 ^a^	36.29 ± 4.11 ^a^	64.08 ± 1.49 ^b^
	PZ19	68.32 ± 3.21 ^ab^	0.96 ± 0.18 ^bc^	17.11 ± 0.45 ^a^	35.73 ± 1.82 ^a^	63.98 ± 2.78 ^b^
	PZ21	70.92 ± 2.39 ^b^	−0.41 ± 0.06 ^a^	13.08 ± 0.47 ^a^	26.32 ± 1.74 ^a^	68.11 ± 2.35 ^c^

YI: yellowness values; WI: whiteness values; Results are expressed as mean ± SD. Values with lowercase letters in common are significantly different (*p* < 0.05).

**Table 3:** Independent weighting analysis of antioxidant activity.

	Standardized	R	1/R	Weight
Brown rice	DPPH	0.689	1.452	21.01
	OH	0.621	1.611	23.32
	ABTS	0.670	1.494	21.62
	FRAP	0.425	2.353	34.05
White rice	DPPH	0.956	1.046	21.31
	OH	0.564	1.772	36.10
	ABTS	0.934	1.071	21.82
	FRAP	0.981	1.019	20.77

R: Complex correlation coefficient.

**Table 4:** Individual phenolic acids and flavonoids intake (mg/sp/day) from white rice and brown rice on a recommended diet.

	White Rice Intake (Approx. 5 Ounces)				Replace 3 Ounces White Rice with Brown Rice				
Samples	Total Phenolic	Total Flavonoids	Total Phenolic Acid	Total	Total Phenolic	Total Flavonoids	Total Phenolic Acid	Total	White/Brown
PZ6	203.1269	257.4621	88.9137	549.5028	251.7561	230.1045	110.8959	592.7565	0.927030
PZ8	165.4938	141.6936	82.4603	389.6478	214.832	198.7454	89.9523	503.5297	0.773833
PZ9	162.3577	238.0754	93.1856	493.6188	232.8222	223.6743	100.9341	557.4306	0.885525
PZ10	188.8874	205.9949	73.6796	468.5619	249.8686	232.7914	115.6612	598.3212	0.783128
PZ12	216.3494	146.1740	96.9800	459.5034	205.4412	189.7433	135.5086	530.6930	0.865855
PZ13	256.2710	179.7998	70.9914	507.0624	225.6261	201.6156	166.6742	593.9159	0.853761
PZ15	262.3737	167.1542	76.8315	506.3595	218.1351	207.0071	139.2290	564.3713	0.897210
PZ19	239.8309	186.1073	68.4853	494.4235	215.1270	191.2810	97.2277	503.6357	0.981709
PZ21	242.8506	155.7384	86.4695	485.0586	251.9485	235.0021	108.4205	595.3711	0.814716

1 ounce equivalent = 28.3 g = 1/2 cup cooked rice.

## Data Availability

The datasets generated for this study are available on request to the corresponding author.
